# Concordance between self-reported SARS-CoV-2 positivity and laboratory-confirmed positivity

**DOI:** 10.1371/journal.pone.0334102

**Published:** 2025-10-17

**Authors:** Collin James Catalfamo, Elizabeth T. Jacobs, Laura P. Falk, Priscilla Lauro, Kacey C. Ernst, Leslie V. Farland, Kelly M. Heslin, Kristen Pogreba-Brown, Pamela C. Garcia-Filion

**Affiliations:** 1 Arizona Research Investigating Disability & Disorders, Department of Pediatrics, College of Medicine – Tucson, The University of Arizona, Tucson, Arizona, United States of America; 2 Mel and Enid Zuckerman College of Public Health, The University of Arizona, Tucson, Arizona, United States of America; 3 Arizona Department of Health Services, Phoenix, Arizona, United States of America; 4 Department of Biomedical Informatics, College of Medicine- Phoenix, The University of Arizona, Phoenix, Arizona, United States of America; Retired-United States Environmental Protection Agency, UNITED STATES OF AMERICA

## Abstract

As the use and availability of at-home antigen tests for SARS-CoV-2 infection have increased, the number of individuals with SARS-CoV-2 infections that are reported to state COVID-19 surveillance systems have decreased. Self-reported infection dates are critical to accurately track incidence and outbreaks of COVID-19 and for continued research on illness progression; however, the reliability of self-reported infection dates is unknown to date. To assess accuracy of self-reported test dates, we utilized self-reported SARS-CoV-2 testing data from the Arizona CoVHORT Study (CoVHORT) and laboratory-confirmed testing data collected by the Arizona Department of Health Services (ADHS) and calculated the difference in days between dates to examine their percent agreement. We used logistic regression to assess if any participant characteristics were associated with self-reporting a test date >7 days different than the laboratory confirmed date. A total of 1,900 CoVHORT participants aged 18 years or older were included in our analyses. Most participants (82.5%) reported a test date within 7 days of the laboratory confirmed date of their illness. Increasing age and number of weeks between testing positive and self-reporting the test date were both significantly associated with a difference of 7 days or greater between dates. There was an 84% increase (OR=1.84, 95% CI = 1.11–3.06) in likelihood of inaccurately self-reporting their SARS-CoV-2 test date for participants aged 55 years and older and a 2% increase (OR=1.02, 95% CI = 1.02–1.03) for each elapsed week following their SARS-CoV-2 test. We observed an 82% percent agreement (dates within 7 days of each other) between self-reported and laboratory confirmed test dates, suggesting that self-reported SARS-CoV-2 test dates are sufficient for identifying and tracking Long COVID or Post-COVID Conditions when a laboratory-confirmed test date is not available. However, increasing age and greater time between test date and date of self-report were found to decrease the agreement between self-reported and laboratory confirmed test dates.

## Introduction

The SARS-CoV-2 pandemic had immense global impacts on morbidity and mortality and has led to extensive societal changes since the first scientific report was published in February 2020 [1]. While early response and research efforts by necessity focused on the impact of acute illness, a collection of longer-term sequelae were identified early in the pandemic and have been further refined over time. These symptoms include cardiovascular abnormalities, gastrointestinal disorders, fatigue, shortness of breath, and “brain fog” or cognitive difficulties [1–14]. Further characterization of long-term symptoms was achieved when the Centers for Disease Control and Prevention defined new or ongoing post-infectious symptoms occurring 4 weeks or more following acute infection as post-COVID condition, or PCC [15,16]. These conditions have also been referred to as post-acute sequelae of COVID-19 (PASC) or, colloquially, as Long COVID [1–14].

To date, numerous studies have been conducted to assess the risk factors for and symptoms of PCC using various epidemiological methods ranging from retrospective medical record reviews to formal prospective studies [5,7–10,14], with many of these studies employing time since self-reported SARS-CoV-2 positivity. This research strategy arose for several reasons, including reducing the burden on study participants, protecting patient privacy, and the increased adoption of home-testing kits. The first of these kits were granted emergency use approval in the United States by the Food and Drug Administration in November 2020 [17]. Uptake of home testing became widespread throughout 2021 and 2022, with the provision of free tests by the United States government under the Biden Administration beginning in January 2022 [18]. Because many studies of acute COVID-19, as well as PCC, rely on accuracy of the positive test date or the date the illness began, it is of key importance to understand the accuracy of self-reported infection dates by comparing them to laboratory-confirmed dates.

Therefore, the primary objective of the present study was to compare self-reported data on testing positive for SARS-CoV-2 with laboratory-confirmed data obtained through COVID-19 surveillance and case investigations conducted by the Arizona Department of Health Services (ADHS). Our secondary objective was to assess whether the accuracy of self-reported positive tests varied by participant characteristics.

## Methods

### Study design and setting

This prospective cohort study analyzed data from participants enrolled in the Arizona CoVHORT Study (CoVHORT) [19] between May 28, 2020, and April 1, 2022. The study was conducted in Arizona using a population-based approach that recruited participants statewide through academic health department partnerships, phased mailing campaigns, and recruitment materials at vaccine distribution sites [20]. Data collection occurred through electronic surveys administered via Research Electronic Data Capture (REDCap) [21], with participants prospectively providing self-reported SARS-CoV-2 testing information at enrollment and follow-up assessments. Self-reported data were subsequently linked to laboratory-confirmed test results from the Arizona Department of Health Services (ADHS) surveillance system (MEDSIS). The analysis period covered SARS-CoV-2 positive test results occurring between January 1, 2020, and April 1, 2022.

### Study participants

Participant information for the present study was collected from two primary sources: CoVHORT and the Arizona Department of Health Services (ADHS) surveillance dataset (MEDSIS), which captures all laboratory-reported positive SARS-CoV-2 test results in the state as part of routine public health surveillance. All labs and hospitals report positive PCR and antigen tests to ADHS which includes the date of testing. We included CoVHORT participants who were 18 years of age or older at enrollment, had self-reported a positive PCR or antigen test date for SARS-CoV-2 infection at baseline or during follow-up, and were enrolled in CoVHORT between May 28, 2020, and April 1, 2022. Laboratory-confirmed test data from MEDSIS included SARS-CoV-2 positive test results that occurred prior to April 2, 2022. For participants with multiple SARS-CoV-2 infections reported to the state surveillance system, only information pertaining to the first reported infection was included in the analysis. We excluded all participants who were missing a self-reported SARS-CoV-2 test or a matched laboratory-reported test.

### Ethical approval

Participants completed all of their surveys electronically via Research Electronic Data Capture (REDCap) [21] and all participants provide written informed consent upon joining the study. The University of Arizona Institutional Review Board provided ethical approval for the completion of the study (Protocol #2003521636).

### Available data

CoVHORT collected information about participant demographics at baseline and assessed SARS-CoV-2 infection status at baseline and during follow-up. Demographic information collected at baseline included age (18−24 years, 25−34 years, 35−44 years, 45−54 years, 55−64 years, or ≥65 years), gender (female or male), race (White, American Indian/Alaska Native, Asian, Black or African American, Native Hawaiian/Pacific Islander, Mixed race, or prefer not to answer), ethnicity (Non-Hispanic, Hispanic, or prefer not to answer), highest level of education completed (High School graduate or less, some college, or college graduate or greater), annual household income (<$25,000, $25,000 to $49,999, $50,000 to $74,999, or ≥$75,000), self-reported employment status (full-/part-time employed, unemployed, or retired), and student status (full-time student or not full-time student). At baseline and during follow-up, participants were asked if they have had a COVID-like illness and/or whether they had tested positive for a SARS-CoV-2 infection, including the type of test used (laboratory conducted polymerase chain reaction (PCR) or antigen tests, or at-home antigen tests) and the date of their test prior to joining the study.

### Case definition

For this study, a “case” was defined as a CoVHORT participant who self-reported a positive SARS-CoV-2 test (PCR or antigen) and had a corresponding laboratory-confirmed positive test recorded in the ADHS surveillance system (MEDSIS). Cases were identified through a data linkage process that matched self-reported positive test dates from CoVHORT participants to their laboratory-reported positive test dates in MEDSIS. The most-likely match between self-reported and laboratory-reported test dates was determined by identifying the pair with the smallest difference in number of days among all potential matches for each participant.

### Outcomes

The primary outcome of this study was to assess the accuracy of self-reported SARS-CoV-2 test dates by comparing participant self-reported positive test dates to laboratory-confirmed PCR or antigen test dates with independent records in the MEDSIS surveillance system. Accuracy was measured by calculating the concordance between self-reported and laboratory-reported test dates, expressed as percent agreement. The difference in days between participant self-reported dates and laboratory-reported dates was used to categorize participants into accuracy groups, with accurate self-reporting defined as test dates differing by seven days or fewer from the laboratory-reported date, and inaccurate self-reporting defined as test dates differing by more than 7 days from the laboratory-reported date.

### Statistical analyses

We first examined concordance between positive test dates reported to MEDSIS and participant self-reported test dates using percent agreement. We calculated the difference, in number of days, between the most-likely self-reported test date match to participants’ laboratory-reported SARS-CoV-2 diagnostic test date. The difference in days between participant self-reported dates and laboratory reported dates were used to categorize participants, and the number and of participants in each group was used to calculate the percent agreement.

Second, we used logistic regression to evaluate factors associated with inaccurate self-reporting of test dates (defined as reporting a test date greater than 7 days different than the laboratory reported date). Independent variables included demographic characteristics (age, gender, race, ethnicity, highest level of education completed, annual household income, self-reported employment status, and student status), symptomatic status at time of diagnostic test for SARS-CoV-2 infection (asymptomatic or symptomatic), and the number of weeks between the date of the diagnostic test and when participants reported the positive test to CoVHORT. We performed univariate logistic regressions to examine the association between self-reporting a test date greater than 7 days different than the laboratory reported test date with each of the independent variables specified. In multivariable models, we included participant age at baseline, with adjustment for number of weeks between the date of the laboratory reported SARS-CoV-2 diagnostic test and the date participants reported their positive test to CoVHORT. Statistical significance was defined as a p-value of 0.05. All analyses were conducted using STATA 18 [22].

## Results

A total of 1900 participants recruited into the CoVHORT study between May 28, 2020, through April 1, 2022, who had a positive laboratory test for SARS-COV-2 reported to MEDSIS between January 1, 2020, and April 1, 2022 (Table 1). The median time between participants’ SARS-CoV-2 laboratory test and the date they reported their test to CoVHORT was 5 weeks (IQR: 1.3 weeks – 16.1 weeks).

**Table 1:** Demographic characteristics of 1,900 Arizona CoVHORT participants aged 18 years or older that enrolled in the study between May 28, 2020, and April 2, 2022, and reported a positive result from laboratory conducted SARS-CoV-2 diagnostic test prior to April 2, 2022.

	Number of participants	Percent of participants
Age (years)		
18-24	234	12.3%
25-34	363	19.1%
35-44	382	20.1%
45-54	346	18.2%
55-64	355	18.7%
≥65	220	11.6%
Gender		
Female	1,323	69.6%
Male	561	29.5%
Non-binary or Transgender	16	0.8%
Race		
American Indian or Alaska Native	29	1.5%
Asian	43	2.3%
Black or African American	21	1.1%
Native Hawaiian or Pacific Islander	<5	–
White	1,660	87.4%
Mixed Race	90	4.7%
Prefer not to answer	42	2.2%
Ethnicity		
Non-Hispanic	1,487	78.3%
Hispanic or Latino/a	373	19.6%
Prefer not to answer	17	0.9%
Highest Level of Education Completed		
High School Graduate or Less	64	3.4%
Some College or Technical School	405	21.3%
College Graduate or Greater	1,100	57.9%
Household Annual Income		
<$25,000	134	7.1%
$25,000 to less than $50,000	224	11.8%
$50,000 to less than $75,000	245	12.9%
≥$75,000 or greater	812	42.7%
Employment status		
Full- or part-time employed	1,416	74.5%
Unemployed	109	5.7%
Retired	226	11.9%
High-risk occupations		
Childcare	12	0.6%
Essential business employee	161	8.5%
Fire Department/EMS	7	0.4%
Healthcare	239	12.6%
Jail/Prison employee	21	1.1%
Law enforcement	17	0.9%
School (K-12)	113	6.0%
Other	132	7.0%
Student status (post K-12)		
Not a full-time student	1,720	90.5%
Full-time student	180	9.5%
Symptomatic at time of testing		
No	259	13.6%
Yes	1,641	86.4%

Overall, 82.5% (n = 1568) self-reported a positive test date within 7 days of the laboratory test date (Table 2). Participant self-reported positive test dates agreed exactly with the laboratory test dates for 28.3% (n = 538) of the cohort. Among participants who did not self-report a positive test date that agreed exactly with the laboratory reported date, most self-reported a positive test date that was after the laboratory test date (46.63% vs 12.53%).

**Table 2:** Percent agreement and number of days between self-reported SARS-CoV-2 diagnostic test dates and laboratory-reported (MEDSIS) test dates among 1,900 Arizona CoVHORT participants aged 18 years or older, who reported a SARS-CoV-2 infection prior to April 2, 2022.

Day difference between MEDSIS date and self-reported date	N	%	Cum. %
<−1 year	5	0.26%	0.26%
−12 to −9 months	10	0.53%	0.79%
−9 to −6 months	4	0.21%	1.00%
−6 to −3 months	9	0.47%	1.47%
−3 to −1 months	17	0.89%	2.37%
−4 to −2 weeks	16	0.84%	3.21%
−14 to −8 days	25	1.32%	4.53%
−7 to −1 days	152	8.00%	12.53%
Exact match	538	28.32%	40.84%
1 to 7 days	878	46.21%	87.05%
8 to 14 days	109	5.74%	92.79%
2 to 4 weeks	43	2.26%	95.05%
1 to 3 months	61	3.21%	98.26%
3 to 6 months	8	0.42%	98.68%
6 to 9 months	8	0.42%	99.11%
9 to 12 months	9	0.47%	99.58%
>1 year	8	0.42%	100.00%

N = number of participants, % = percent of participants, Cum. % = cumulative percent of participants.

Table 3 presents the likelihood of disagreement by participant characteristics. For every ten years of age, the odds of disagreement (>7 days difference) increased by 15% (OR 1.15). Individuals who self-reported their employment status as being retired were observed to have a 50% increase in the odds of disagreement (OR 1.50). Those aged 55–64 years (OR 2.12) and 65 + years (OR 1.96) had approximately two-fold odds of disagreement compared to 18–24-year-olds. These associations of age persisted after adjusting for number of weeks since testing positive to reporting illness to CoVHORT (Table 4).

**Table 3:** Unadjusted odds ratios (95% confidence intervals) for self-reporting a SARS-CoV-2 diagnostic test date to the Arizona CoVHORT greater than 7 days different than the matched laboratory test date reported to ADHS.

Variable	uOR	95% CI	p
Age (every 10 years)	1.15	(1.07, 1.24)	<0.001
18-24 years	ref		
25-34 years	1.18	(0.73, 1.92)	0.501
35-44 years	1.40	(0.87, 2.24)	0.167
45-54 years	1.42	(0.88, 2.30)	0.150
55-64 years	2.12	(1.34, 3.37)	0.001
≥65 years	1.97	(1.19, 3.26)	0.008
Gender			
Female	ref		
Male	1.08	(0.83, 1.40)	0.565
Race			
White	ref		
American Indian or Alaska Native	1.20	(0.48, 2.97)	0.697
Asian	0.89	(0.39, 2.02)	0.785
Black or African American	1.08	(0.36, 3.23)	0.891
Native Hawaiian or Pacific Islander	–	–	–
Mixed race	0.64	(0.34, 1.22)	0.172
Prefer not to answer	0.76	(0.32, 1.83)	0.547
Ethnicity			
Non-Hispanic	ref		
Hispanic	0.83	(0.60, 1.13)	0.232
Prefer not to answer	1.44	(0.47, 4.45)	0.528
Highest Level of Education Completed			0.757a
High School Graduate or Less	0.89	(0.45, 1.78)	0.748
Some College or Technical School	1.11	(0.83, 1.49)	0.475
College Graduate or Greater	ref		
Household Annual Income			0.028a
<$25,000	0.67	(0.40, 1.13)	0.132
$25,000 to less than $50,000	0.68	(0.45, 1.03)	0.066
$50,000 to less than $75,000	0.84	(0.58, 1.23)	0.372
≥$75,000 or greater	ref		
Employment status			
Full- or part-time employed	ref		
Unemployed	0.91	(0.53, 1.55)	0.716
Retired	1.50	(1.07, 2.10)	0.018
Student status (post K-12)			
Not a full-time student	ref		
Full-time student	0.60	(0.37, 0.96)	0.033
Symptomatic at time of testing			
No	ref		
Yes	0.87	(0.62, 1.21)	0.404
Number of weeks between testing positive and reporting illness to CoVHORT	1.02	(1.02, 1.03)	<0.001

95% CI = 95% confidence interval, uOR = unadjusted odds ratio, p = p-value, *ref* = referent group

^a^p-value of test for trend

**Table 4:** Adjusted odds ratios (95% confidence intervals) for self-reporting a SARS-CoV-2 diagnostic test date to the Arizona CoVHORT greater than 7 days different than the matched laboratory test date reported to ADHS.

Variable	aORa	95% CI	p
Age (every 10 years)			
18-24 years	ref		
25-34 years	1.12	(0.69, 1.84)	0.636
35-44 years	1.27	(0.79, 2.06)	0.324
45-54 years	1.28	(0.79, 2.08)	0.322
55-64 years	1.85	(1.16, 2.96)	0.010
≥65 years	1.84	(1.11, 3.06)	0.019
Number of weeks between testing positive and reporting illness to CoVHORT	1.02	(1.02, 1.03)	<0.001

95% CI = 95% confidence interval, aOR = adjusted odds ratio, p = p-value, *ref* = referent group.

^a^Adjusted logistic regression model included adjustment for age (when primary exposure was number of weeks between testing positive and reporting illness), and adjustment for number of weeks between testing positive and reporting illness to CoVHORT (when age was the primary exposure).

Disagreement became more likely with increasing time intervals (number of weeks) between infection status and reporting illness to CoVHORT (OR 1.02)(Table 4); this association remained unchanged after adjusting for age. Testing positive four weeks prior to reporting the illness to CoVHORT was associated with a 9.4% increased likelihood of an inaccurate self-report of test dates, and an interval of three months was associated with a 30.8% increased likelihood.

## Discussion

The results of this study show that the vast majority of participants in a population-based cohort study can accurately self-report their laboratory test date. Approximately 82% of participants reported a test date that was within 7 days of the laboratory-confirmed date collected by the state health department, 89% were within 14 days, and 92% were within 28 days. Those aged 55 years or above or reported being retired were significantly more likely to self-report dates that differed by 7 days or more from laboratory-confirmed testing than younger groups and those who are currently employed at work or as a student. Finally, a longer length of time between testing positive and completing the survey was significantly and inversely associated with the accuracy of test date reporting.

Participant responses were less accurate among participants aged 55 years and above as compared to younger respondents. This finding has been demonstrated in prior studies of the impact of age on survey responses [23]. The accuracy of self-reports requires several aspects of cognitive processes, including understanding survey items, recall ability, and information processing [23]. In addition, individuals with frequent healthcare utilization may face additional challenges in distinguishing between multiple similar medical encounters, potentially increasing variability in recall of specific test dates and medical events [24–26]. Despite the variation in report accuracy by age, a stronger relationship was observed for the length of time between the laboratory-confirmed test date and the date of self-report, which supports prior work. For example, it was demonstrated that monthly self-reports of healthcare utilization effects greater accuracy regarding healthcare utilization than do yearly surveys [24], indicating that reports which are more proximal to the events being queried are more reliable. This phenomenon was first described in detail by Ebbinghaus in 1885, has been supported by a wealth of studies related to memory capacity, and arises due to numerous cognitive processes [27]. Hence, regular administration of surveys regarding COVID-19 test dates, as with the majority of other self-reported data, yield benefits in regard to accuracy of reporting.

Overall, we observed moderately high accuracy between the participant self-reported diagnostic test dates and those that were reported to ADHS by the laboratories that processed participant samples, with 82.5% of participants reporting a date within 7 days of the laboratory reported date. Although, slightly less accurate, our results are similar to other studies examining the reliability and accuracy of self-reported COVID vaccination status and other conditions. Among patients diagnosed with a SARS-CoV-2 infection who visited Canadian emergency departments, 96% of patients correctly reported their vaccination status at telephone follow-up compared to their vaccination record as in the Quebec Vaccination Registry [28]. A separate study conducted among adolescent girls further supports the reliability of self-reported diagnoses, which found a high overall agreement between self-report, medical record, and state surveillance record for past chlamydia and gonorrhea diagnoses [29].

Much about the acute-illness and long-term sequelae remain unknown despite the numerous studies of COVID-19 that have been conducted since the first appearance of SARS-CoV-2. Accurate infection dates, symptom start dates and dates of diagnosis, are crucial to ongoing research of illness progression, including PCC. However, with the widespread availability and utilization of at-home antigen tests, an increasing proportion of COVID-19 cases are likely not reported to health department surveillance systems and therefore are not associated with any laboratory confirmed testing information. The reliability of self-reported infection dates observed in this study support their utilization in future research, including those associated with at-home tests that have not been previously reported to any health-department based surveillance system.

Strengths of this study include the ability to collaborate with the Arizona Department of Health Services to obtain accurate data for laboratory testing dates, a large study population, and well-characterized participant data that permitted the evaluation of characteristics that were related to accuracy of testing self-reports. Limitations include a largely white and highly educated study population, affecting the generalizability of these findings.

In conclusion, these findings demonstrate that self-reported COVID-19 test dates exhibit high concordance with laboratory-based test dates reported to the state health department in Arizona. These findings should provide confidence in using these data to assess the presence or absence of PCC among individuals who have tested positive for SARS-CoV-2.
